# Effectiveness of a Household Water, Sanitation and Hygiene Package on an Outpatient Program for Severe Acute Malnutrition: A Pragmatic Cluster-Randomized Controlled Trial in Chad

**DOI:** 10.4269/ajtmh.17-0699

**Published:** 2018-02-26

**Authors:** Mathias Altmann, Chiara Altare, Nanette van der Spek, Jean-Christophe Barbiche, Jovana Dodos, Mahamat Bechir, Myriam Ait Aissa, Patrick Kolsteren

**Affiliations:** 1 Action Contre la Faim, Paris, France;; 2 Institute of Tropical Medicine, Antwerpen, Belgium;; 3 Alliance Sahélienne de Recherches Appliquées pour le Développement Durable, Quartier Klemat, N’Djamena, Chad;; 4 Gent University, Gent, Belgium

## Abstract

Water, sanitation and hygiene (WASH) interventions have a small but measurable benefit on stunting, but not on wasting. Our objective was to assess the effectiveness of a household WASH package on the performance of an Outpatient Therapeutic feeding Program (OTP) for severe acute malnutrition (SAM). We conducted a cluster-randomized controlled trial embedded in a routine OTP. The study population included 20 health centers (clusters) from Mao and Mondo districts in Chad. Both arms received the OTP. The intervention arm received an additional household WASH package (chlorine, soap, water storage container, and promotion on its use). The primary objective measures were the relapse rates to SAM at 2 and 6 months post-recovery. The secondary objectives included the recovery rate from SAM, the time-to-recovery, the weight gain, and the diarrhea longitudinal prevalence in OTP. The study lasted from April 2015 to May 2016. Among the 1,603 recruited children, 845 were in the intervention arm and 758 in the control arm. No differences in the relapse rates were noticed at 2 (−0.4%; *P* = 0.911) and 6 (−1.0%; *P* = 0.532) months. The intervention decreased the time-to-recovery (−4.4 days; *P* = 0.038), improved the recovery rate (10.5%; *P* = 0.034), and the absolute weight gain (3.0 g/d; *P* = 0.014). No statistical differences were noticed for the diarrhea longitudinal prevalence (−1.7%; *P* = 0.223) and the weight gain velocity (0.4 g/kg/d; *P* = 0.086). Our results showed that adding a household WASH package did not decrease post-recovery relapse rates but increased the recovery rate among children admitted in OTP. We recommend further robust trials in other settings to confirm our results.

## INTRODUCTION

It is estimated that 58% of annual deaths caused by diarrhea are attributable to poor water, sanitation and hygiene (WASH) conditions.^1^ Interventions aiming at improving water quality at household level^2,3^ or at promoting hand washing with soap significantly reduce diarrhea incidence.^4,5^ WASH interventions have a small but measurable benefit on linear growth, but not on weight or weight-for-height.^6^ Improved nutrition can reduce the adverse effects of infections such as diarrhea on growth.^7^ Yet, little evidence exists as to whether infections hamper the effectiveness of nutrition interventions^7^ and whether combined nutrition and WASH interventions would be more effective.^8^

To increase synergies in the fight against acute malnutrition, WASH and nutrition actors led by United Nations International Children’s Fund (UNICEF) and European Civil Protection and Humanitarian Aid Operations, agreed in 2012 on a “WASH in Nut” strategy for the Sahel region.^9^ This strategy targets communities and health centers (HCs) in areas with high prevalence of acute malnutrition. Interventions in HCs include the provision of a household WASH package designed to protect children against new episodes of diarrhea and aiming at improving nutritional outcomes.

In Chad, undernutrition remains a public health concern. In the Kanem region (northwest of the country), the prevalence of acute malnutrition was reported beyond emergency thresholds in 2014 (17.3% global acute malnutrition and 3.5% severe acute malnutrition [SAM]).^10^ In 2013, the Kanem nutrition rehabilitation program admitted around 11,000 children for noncomplicated SAM (73% were successfully treated, 10% did not respond to the treatment, 7% were transferred to hospital or died, 10% abandoned the program, and more than 20% of total admissions were children who relapsed from a previous cured SAM episode). Besides acute malnutrition, diarrheal diseases represented the largest notified disease among children (32% of the HC admissions). The prevalence of intestinal parasitic infection is reported to be 60% (95% confidence interval [CI] = 53–66), and worm infestation has been attributed to water quality.^11^

Against this backdrop, we hypothesized that nutrition rehabilitation of SAM could benefit from a complementary WASH intervention at the household level. Improving water quality and hygiene-related care practices at household level would decrease the incidence of diarrhea during the Outpatient Therapeutic feeding Program (OTP), and consequently, improve recovery, daily weight gain, and shorten the children’s time-to-recovery. Furthermore, it was expected that in the months after discharge from OTP, the improvement of WASH-related practices would decrease the risk of WASH-related infections. This would possibly improve the child’s immune recovery and reduce the risk of relapsing after successful discharge.

To our knowledge, no WASH intervention has been assessed so far, either as a complement to a nutrition rehabilitation program,^12^ or after discharge when immune recovery is still incomplete.^13^ Therefore, we investigated the additional benefits of a WASH intervention among 6- to 59-month-old children admitted to an OTP in the Kanem region in Chad. The primary outcome measures were the relapse rates at 2 and 6 months post-recovery. Secondary outcomes included recovery rate, time-to-recovery, and weight gain and diarrhea longitudinal prevalence at OTP discharge.

## MATERIALS AND METHODS

### Study design and participants.

This study was conducted in Mondo and Mao districts, Kanem region, Chad. The design was a cluster-randomized controlled trial embedded in a routine nutritional program for outpatient SAM management supported by the international non-governmental organization Action Contre la Faim (ACF). Health centers were the unit of randomization. Twenty of the 35 existing HC in the study area were included in the study based on their location (they were not contiguous from each other to avoid contamination among participants) and their performance (top scores in terms of quality and availability of services as measured by ACF’s performance self-evaluation tool). All new admissions without complications were eligible to the study. Children transferred from other HC, where their treatment had already started, were excluded. Routine criteria for OTP admission included children aged between 6 and 59 months with a weight-for-height *Z* (WHZ) score of < −3 and/or a mid-upper-arm-circumference (MUAC) of < 115 mm, and/or the presence of mild or moderate bilateral edema. Children with signs of medical complications requiring inpatient management or severe bilateral edema were not included in the study and referred to the nearest hospital for inpatient management after agreement of the caretaker.

### The intervention.

Both groups received OTP services as routinely implemented (as per the national guideline for nutrition rehabilitation)^14^ and basic hygiene education and care practice sessions during HC visits. Intervention clusters also received the household WASH kit at admission. This contained a safe drinking water storage container with a lid, water disinfection consumables (180 chlorine tablets), 12 bars of soap for hand washing, a plastic cup with handle (to be reserved for the child to facilitate safe drinking water practice), and a laminated leaflet with pictures representing the main hygiene messages. They also participated to a promotion session on the kit use at each weekly visit to the HC and two extra home visits for assessing and reinforcing adherence. Specifically, promotion at HC included key messages on 1) a protected space for children to play; 2) washing the child with soap; 3) cleaning and rapid burial of children’s stools; 4) hand washing at key times; 5) safe storage of water; 6) exclusive breastfeeding of children before 6 months; and 7) water treatment and food hygiene. The household WASH kit was designed to last for 3 months (2 months during treatment in the OTP and 1 month after the end of the treatment).

The amount of chlorine tablets (Aquatab^®^; Hydrachem Ltd, Billlingshurst, United Kingdom) given was based on the need to purify around 40 L of water per family per day (two tablets). The number of soap bars corresponded to 250 grams per person per month for the mother and her child plus 250 grams per month for the rest of the family sufficient to cover a 3-month period. As compensation, the control group received a loincloth at the end of the OTP phase.

### Outcomes.

Primary outcome was the relapse rate to SAM. “Relapse” was defined as a new SAM event (MUAC < 115 or WHZ score < −3 or bilateral pitting edema) during home visits at 2 or 6 months after a successful discharge. Relapsing at 2 months was considered as a censoring event and these children were not followed up at the 6-month visit.

Secondary outcome measures included the recovery rate from SAM, the time-to-recovery, the weight gain and the diarrhea longitudinal prevalence at OTP discharge. Recovery rate was assessed based on whether a child was cured at discharge. Depending on the admission criteria, being cured was defined as either achieving a MUAC of 125 mm or a WHZ score of ≥ −2 taking length at admission, with no presence of bilateral edema in the previous 14 days. It was noticed during the analysis, that in the routine OTP practice, anthropometric criteria for discharge were not always strictly applied and discharge defined as “cured” was decided based on clinical appraisal before anthropometric normalization. Poor adherence to the protocol could have introduced a misclassification bias. To assess this potential bias on the recovery rate, we performed a sensitivity analysis by defining children as cured strictly according to their anthropometric measurements (and not to their discharge status).

Daily weight gain velocity was calculated for cured children according to the following formula:

$$\text{daily}\;\text{weight}\;\text{gain}\;\text{velocity}=\frac{\text{weight}\;\text{at}\;\text{discharge}-\text{weight}\;\text{at}\;\text{admission}\left(\text{gram}\right)}{\text{weight}\;\text{at}\;\text{admission}\left(\text{kilogram}\right)\times \text{length}\;\text{of}\;\text{stay}\left(\text{days}\right)}.$$

In addition, absolute weight gain was calculated using weight at discharge minus weight at admission (in gram) as numerator and length of stay (days) as denominator. Length of stay (days) was calculated only for cured children. Diarrhea was defined as having loose or watery stools at least three times per day during the week before the visit at the HC during the OTP phase.

Other possible outcomes at discharge were “defaulter” (when the child was absent during two consecutive visits), “died,” “transferred to a stabilization center,” or “nonresponder” (children who did not gain weight and should be referred but caretakers refused to go to hospital). The number of days with vomiting, fever, and cough were based on recall of the mother during the week before the visit during the OTP phase.

Hygiene knowledge and related care practices were evaluated through a knowledge, attitude and practice survey using standardized questions based on self-reporting. A WASH score (ranging from 0 to 8) was calculated by assessing eight behaviors of the child’s caretaker (one point for a correct and 0 for an incorrect behavior). The eight WASH behaviors included 1) child’s body hygiene; 2) fecal contamination–free child play areas; 3) safe disposal of child’s faces; 4) household drinking water treatment; 5) transport of water from the source to the point-of-use; 6) safe water storage; 7) knowledge of the key times for hand washing; and 8) food hygiene.

### Randomization and masking.

Health centers were stratified in pairs (intervention and control) according to the monthly number of SAM admissions (historic data from the year 2013) to obtain a balanced number of enrolments in the two arms. We randomly extracted one letter of the alphabet and we assigned within each pair the intervention to the HC with the first letter of its name closest to this letter. Masking of participants was not possible because of the nature of the intervention. To limit judgment bias, the research and HC staff members were different. Whereas the HC staff collected nutritional and morbidity data in OTP, research staff was responsible of collecting WASH data. It was not possible to blind research staff, but they rotated so they covered different groups. Recruitment started 1 month after allocation of each HC to either group.

### Sample size.

Program data indicated that 20% of cases were relapse cases at admission but the relapse rate post-recovery remained unknown in the study area. Based on other studies,^14,15^ we estimated a cumulative true relapse rate of 12% during the 6 months after successful discharge. Furthermore, we estimated a coefficient of variation of the true rate between clusters within each group (*k*) of 0.17 based on the number of admitted cases in the 20 clusters as reported in program data in 2013. Using the formula presented by Hayes et al.,^16^ an alpha error of 5% and a beta error of 20%, we needed 1,400 cured children from 20 clusters to detect a 5% difference between the two groups.

### Data collection.

The study recruitment phase lasted from April to December 2015 and the follow-up phase finished by May 2016. Ten field monitors were recruited locally and received a technical training in Mao on appropriate data collection, water quality tests, and how to promote the WASH kit. The HC staff members as part of their routine procedures collected weekly data on sociodemographic characteristics, nutritional status, morbidity, and treatment. Height was measured to the nearest 1 mm (UNICEF measuring scale) and weight was measured to the nearest 100 grams (calibrated Salter hanging scale for children weighing 10 kg or more and a calibrated electronic scale (type: UNISCALE) for children weighing less than 10 kg). Mid-upper-arm-circumference was measured to the nearest 1 mm using an arm circumference measuring tape. Height-for-age *Z* (HAZ) scores and WHZ scores were calculated based on the UNISEX *Z* scores table, as used by the Chad Ministry of Health.^17^ Water, sanitation and hygiene variables were collected by the field monitors at the HC through a standardized questionnaire, at admission and discharge. During the OTP phase, two home visits in the intervention group aimed to measure water quality (by turbidimetry and presence of chlorine residual), to collect information on the adherence to the WASH package, and to provide extra promotion session on the usage of the WASH kit. In the study area, chlorine was not available, and therefore, no data were collected in the control group. Turbidity was measured with a two-part turbidity tube to assess the suitability of the chlorine intervention. Free chlorine (residual) was measured with a pooltester with a detection limit of 0.1 mg/L. According to ACF standards,^18^ correct turbidity and correct chlorine residual were defined between 0 and 20 nephelometric turbidity units and ≥ 0.5 mg/L, respectively. Acceptance of chlorinated water was self-reported by the mother and defined as “at least the child drinks the chlorinated water.” In addition, cured children from both groups received home visits 2 and 6 months after OTP discharge to assess whether the child relapsed and to collect data on hygiene knowledge and related care practices. All collected forms were inserted in an open data kit database with integrated validity checks. Data were double entered by two different data managers and checked for consistency and accuracy by the project manager.

### Statistical analysis.

All analyses were conducted in STATA 13 (Stata Corp, College Station, TX) on an intention-to-treat basis by using the full dataset. Statistical significance was set at < 5% with a two-sided test. For anthropometric values, World Health Organization standards with recommended flag limits were used (±6 standard deviations [SD] for HAZ and ±5 SD for WHZ.^19^ We compared categorical and continuous variables at baseline with clustered χ^2^ tests and clustered *t* tests, respectively (clustering was at HC level). Kaplan–Meier multilevel survival analysis was used to display the length of stay, with time-to-recovery as censoring event and a maximum stay of 12 weeks. Multilevel mixed-effects linear regression models at individual level were used for all outcomes, with a random effect for clusters to account for the clustered design.^20^ All models were adjusted for the number of missed visits (no treatment because of ready-to-use therapeutic foods shortage), WHZ score at admission, gender, and age of the child. We ignored the pairing in our analysis as it has been shown to be valid and efficient when trials have small number of relatively large clusters.^21^ Outcomes were presented as means with their standard errors for continuous outcomes and as rates for binary outcomes estimated from the models. Intervention effects were presented as absolute differences with their 95% CI. Longitudinal prevalence for morbidity outcomes was only measured during the time that the child was in OTP and defined as proportion of time under observation with the disease.^22^ Total morbidity was calculated by summing up all symptom days.

### Ethics.

The trial was registered at clinicaltrials.gov under the identifier: NCT02486523 and approved by the General Secretary of the Ministry of Public Health in Chad and the institutional ethical review board of the Institute of Tropical Medicine in Antwerp. The consolidated standards of reporting trials guidelines were followed for this report.^23^ An oral informed consent was obtained from all caretakers whose child was eligible for the study.

## RESULTS

**Figure 1.**
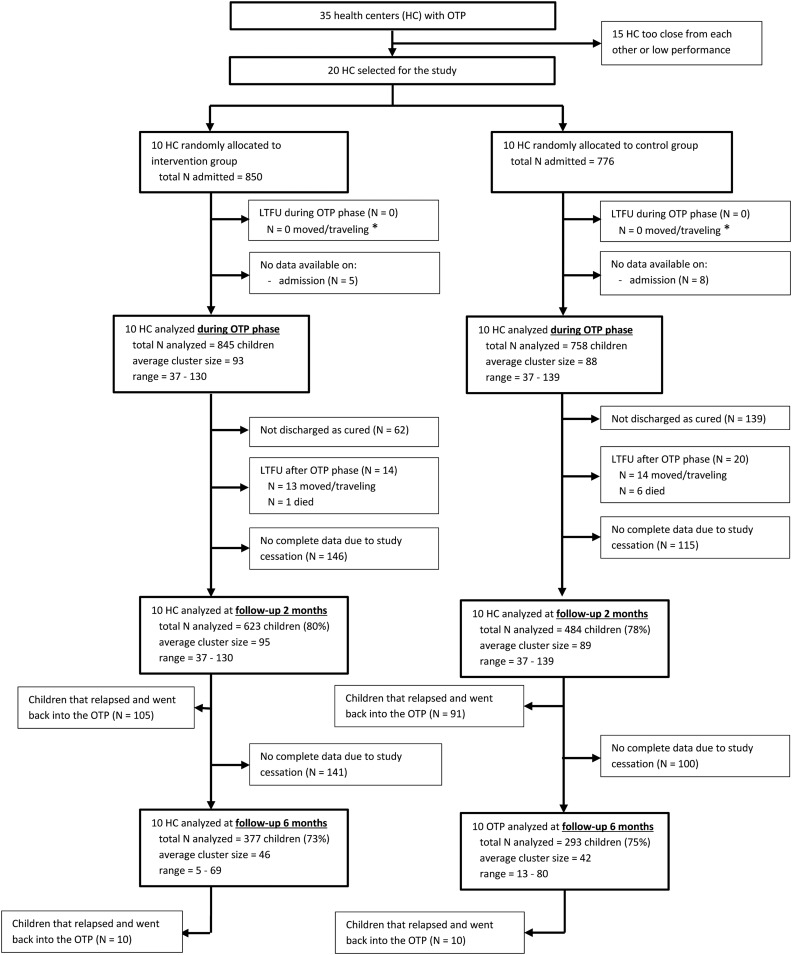
Flowchart of allocation, follow-up, and analysis of the data. * Two children of the intervention group and four children of the control group died during the OTP phase; however, death rate is an outcome, so these children will be analyzed. HC = health center; LTFU = lost to follow-up; OTP = Outpatient Therapeutic feeding Program.

A total of 1,616 children between 6 and 59 months of age were recruited from 20 HC (Figure 1), of which 13 were excluded due to incomplete data at admission. Among the 1,603 children, 845 were in the intervention group and 758 in the control group. Two months after discharge, we followed up on 623 and 484 children (80% and 78% of cured children) in the intervention and the control group, respectively. Six months after discharge, 377 and 293 children remained in the intervention and the control group, respectively (corresponding to 73% and 75% of the children who did not relapse 2 months after discharge).

Table 1 shows baseline characteristics of study participants by intervention and control groups. Sociodemographic characteristics were not significantly different between the two groups as for gender, age, breastfeeding, familial status of the caretakers, number of people within the household, and number of children < 5 years per household, and distance from HC to home. Nutritional status of children at baseline was also similar. Around 61% of admitted children were severely stunted. Proportions of severely wasted were 75.8% and 61.1% based on WHZ score and MUAC, respectively. Some differences were noticed between the groups, but these differences should have happened by chance. Children in the control group had more up-to-date vaccination status.

**Table 1:** Baseline characteristics of intervention and control participants

Characteristics	Intervention		Control	
	*n*/*N*	%	*n*/*N*	%
Gender, male	359/845	42.5	320/758	42.2
Age, 6–23 months	536/845	63.4	516/758	68.0
Presently breastfed	466/844	55.2	450/758	59.4
Caretaker, mother	824/845	97.5	735/756	97.2
Household size—*N*, mean (SD)	841	5.0 (2.0)	752	4.8 (1.4)
Number of children U5 in the household—*N*, mean (SD)	841	1.7 (0.6)	752	1.6 (0.7)
Distance from health center to home (minute)—*N*, median (IQR)	836	60 (30)	749	50 (30)
Stunted				
HAZ score—*N*, mean (SD)	803	−3.3 (1.5)	713	−3.3 (1.5)
Not stunted	142/803	17.7	126/713	17.7
Stunted (−3 SD < HAZ < −2 SD)	182/803	22.7	144/713	20.2
Severely stunted (HAZ < −3 SD)	479/803	59.6	443/713	62.1
Wasted based on *Z* scores*				
WHZ score—*N*, mean (SD)	795	−3.3 (0.8)	717	−3.4 (0.7)
Not wasted	45/795	5.7	41/717	5.7
Wasted (−3 SD < WHZ score < −2 SD)	151/795	19.0	129/717	18.0
Severely wasted (WHZ score < −3 SD)	599/795	75.3	546/717	76.3
Wasted based on MUAC				
MUAC—*N*, mean (SD)	845	114 (7.2)	735	113 (7.5)
Not wasted	52/845	6.2	38/735	5.2
Wasted (115 < MUAC < 125)	300/845	35.3	224/735	30.5
Severely wasted (MUAC < 115)	493/845	58.3	473/735	64.3
Type of admission				
New admission	784/845	92.8	730/758	96.3
Transfer from stabilization center	1/845	0.1	2/758	0.3
Relapse	60/845	7.1	26/758	3.4
Morbidity of the child				
Diarrhea	274/845	32.4	172/758	22.7
Vomiting	35/845	4.1	42/758	5.5
Fever	94/845	11.1	85/758	11.2
Cough	209/845	24.9	101/758	13.3
Conjunctivitis	128/845	15.2	180/758	24.8
Edema				
No edema (−)	827/845	97.9	747/755	98.6
Mild edema (+)	12/845	1.4	8/755	1.1
Moderate edema (++)	6/845	0.7	3/755	0.4
Vaccination card	190/778	24.4	209/668	31.3
Vaccination up-to-date	372/781	47.6	399/684	58.3
Amoxicillin at admission	692/823	84.1	657/741	88.7

IQR = interquartile range; HAZ = height-for-age *Z*; MUAC = mid-upper-arm-circumference; SD = standard deviation; WHZ = weight-for-height *Z*.

Table 2 presents the intervention effects on primary, secondary, and tertiary outcome measures. Relapse rate was higher at 2 than 6 months post-recovery, with around 18% and 3%, respectively. No statistical difference was noticed between the groups in the relapse rates, both at 2 and 6 months post-recovery.

**Table 2:** Intervention effectiveness on primary, secondary, and tertiary outcomes

Outcomes	Intervention		Control		Intervention effect		
					Absolute difference	95% CI	*P* value
Primary outcome measure							
Relapse rate at 2 months, *n*/*N*, %	105/623	17.6	91/484	18.0	−0.4	[−7.2; 6.4]	0.911
Relapse rate at 6 months, *n*/*N*, %	10/377	2.6	10/293	3.6	−1.0	[−4.0; 2.0]	0.532
Secondary outcome measures							
Recovery rate (program), *N*, %	783/845	92.4	618/758	81.9	10.5	[6.7; 19.8]	0.034
Recovery rate (sensitivity analysis), *N*, %	675/845	79.4	521/758	69.8	9.6	[6.7; 19.8]	0.043
Time-to-recovery in days (program), *N*, mean ± SE	783	51.7 ± 1.5	618	56.1 ± 1.5	−4.4	[−8.6; −0.2]	0.038
Time-to-recovery in days (sensitivity analysis), *N*, mean ± SE	675	52.9 ± 1.5	521	56.6 ± 1.5	−3.7	[−7.8; 0.4]	0.075
Weight gain velocity (g/kg/d), *N*, mean ± SE	783	4.2 ± 0.2	618	3.8 ± 0.2	0.4	[−0.05; 0.8]	0.086
Absolute weight gain (g/d), *N*, mean ± SE	783	27.5 ± 0.8	618	24.5 ± 0.9	3.0	[0.6; 5.4]	0.014
Diarrhea, *N*, LP ± SE	844	1.5 ± 1.0	749	3.2 ± 1.0	−1.7	[−4.5; 1.0]	0.223
Tertiary outcome measures							
Defaulter rate, *n*/*N*, %	35/845	4.5	36/758	4.8	−0.3	[−3.9; 3.3]	0.880
Internal transfer rate, *n*/*N*, %	8/845	0.9	7/758	0.9	0.0	[−0.1; 0.1]	0.969
Death rate, *n*/*N*, %	2/845	0.2	4/758	0.5	−0.3	[−0.9; 0.3]	0.361
Nonresponder rate, *n*/*N*, %	17/845	2.0	93/758	11.7	−9.7	[−16.9; −2.4]	0.009
Vomiting, *N*, LP ± SE	844	0.1 ± 0.1	749	0.6 ± 0.1	−0.5	[−0.9; −0.06]	0.023
Cough, *N*, LP ± SE	844	0.5 ± 0.3	749	0.9 ± 0.3	−0.5	[−1.2; 0.3]	0.213
Fever, *N*, LP ± SE	844	0.2 ± 0.3	749	0.8 ± 0.3	−0.6	[−1.5; 0.2]	0.159
Total morbidity, *N*, LP ± SE	844	2.2 ± 1.2	749	5.4 ± 1.3	−3.2	[−6.7; 0.2]	0.066

CI = confidence interval; LP = longitudinal prevalence; SE = standard error; WHZ = weight-for-height *Z*.

* All models were adjusted for age, gender, WHZ score at baseline, and number of missed visits because of RUTF shortage.

The recovery rate was 10.5% higher in the intervention group compared with the control group, with 92.4% and 81.9% of the cases, respectively (*P* = 0.034). In the sensitivity analysis, despite a lower absolute recovery rate, the effect of the WASH intervention did not change (9.6% absolute difference between the two groups: 79.4% versus 69.8%; *P* = 0.043).

**Figure 2.**
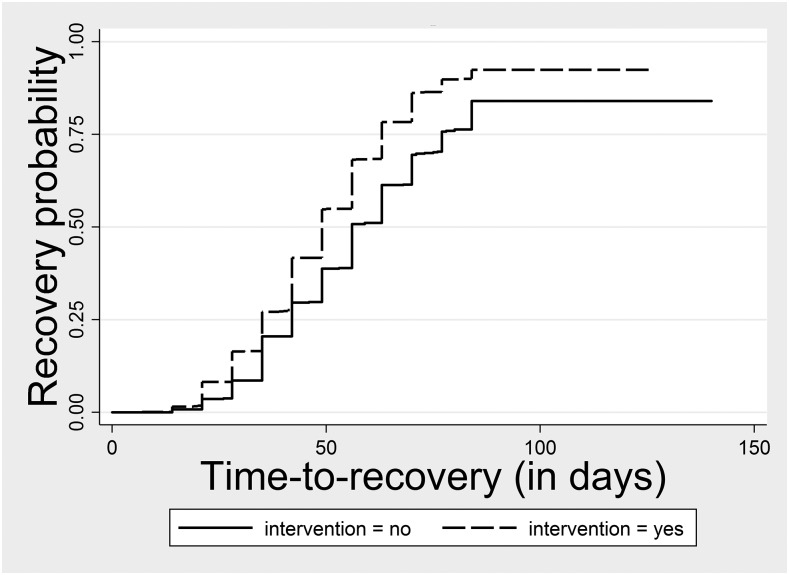
Time-to-recovery per intervention and control group, Mao and Mondo health districts, 2015–2016.

Time-to-recovery was 4.4 days shorter in the intervention group (51.7 days) compared with the control group (56.0 days) (*P* value = 0.038). Survival functions show that time-to-recovery curves diverge (Figure 2) over the treatment duration when recovery should be achieved (maximum of 12 weeks/84 days as per national protocol). In the sensitive analysis, the time-to-recovery was 3.7 days shorter in the intervention group, but not statistically different. The weight gain velocity was 0.4 g/kg/d higher in the intervention group compared with the control group, but this difference was not statistically significant (*P* value = 0.086). Absolute weight gain was 3 g/d significantly higher in the intervention group (*P* value = 0.014). Diarrhea longitudinal prevalence was low in both groups and not statistically different.

The rates of the other types of discharge did not differ between the groups, except for the nonresponder rate, which was lower in the intervention group (*P* = 0.009). Compared with the control group, the longitudinal prevalence of reported symptoms was lower in the intervention group, although not statistically different, except for vomiting (*P* = 0.023).

During the OTP phase, shortages of RUTF affected similarly the two groups (2.9 and 2.6 times per child in the control and intervention groups, respectively; *P* = 0.6695). Each visit with a shortage in RUTF was associated with a 2.5% reduction in the recovery rate (*P* = 0.000) and with a 0.2% increase in the transfer rate (*P* = 0.039).

Adherence to the WASH package was assessed two times in the intervention group, during the first and the second month of the nutritional rehabilitation. Table 3 describes the proportions of correct use of the different components of the WASH kit. The correct procedure to transport the water improved from 47.9% to 65.3% between the two visits. Use of chlorine and its reported correct utilization improved from around 50 to 60% and from 67.6% to 83.7%, respectively. The water turbidity ranged correctly for about 87% of the households, indicating that water source was not turbid in the area and that Aquatab could work. Proportion of residual chlorine equal or greater than 0.5 mg/L in household water decreased from around 60 to 50% between the two visits, but only 1–2% had no trace of chlorine. Acceptance of chlorinated water improved over time, with around 65% and 79% at the first and the second home visit, respectively. Correct use of storage container improved but did not exceed 50%. The procedure for handwashing was correctly applied in 65% of the households.

**Table 3:** Adherence to the WASH kit in the intervention group

Indicator	First visit		Second visit	
	*n*/*N*	%	*n*/*N*	%
Correct procedure to transport the water	340/710	47.9	448/686	65.3
Use of Aquatab: water always chlorinated	351/710	49.4	420/686	61.2
Correct chlorination procedure	480/710	67.6	574/686	83.7
Correct turbidity analysis (0–20 NTU)	620/712	87.1	604/690	87.6
Correct residual chlorine present in the water (> 0.5 mg/L)	283/696	60.7	343/677	50.7
Acceptance of chlorinated water	461/710	64.9	540/686	78.8
Correct use of storage container	203/710	28.6	342/686	49.9
Correct procedure for handwashing	307/709	43.3	446/686	65.0
Laminated leaflet present in household and used	673/707	95.2	658/686	95.9

WASH = water, sanitation and hygiene.

The WASH score was not significantly different at admission between the two groups, with a mean of around five points on a scale of 8 (absolute difference = −0.3; 95% CI = [−0.7; 0.2]; *P* = 0.246). Both groups improved their WASH-related practices from admission to discharge but the increase was statistically higher in the intervention group (absolute difference = 1.0; 95% CI = [0.6; 1.4]; *P* = 0.000). After discharge, whereas the WASH score remained stable in the control group, it decreased in the intervention group and became not statistically different from the control group at 6-month follow-up.

## DISCUSSION

Our study aimed to assess the effectiveness of a household WASH package on the recovery and relapse rates of SAM children admitted to OTP in the Kanem region of Chad. To our knowledge, this is the first study to investigate the additional benefits of a WASH intervention on a nutrition rehabilitation program in Sahel. Studies published so far were conducted in the general population and did not aim primarily to assess the effect on recovery and relapse rates.^6^ Our results showed that adding a household WASH package enhanced program performance by increasing the recovery rate and, possibly, by decreasing the time-to-recovery. Relapse rates to SAM were not affected by the intervention either at 2 or at 6 months post-recovery.

The WASH intervention did not reduce the relapse rate between the groups. This may be partially explained by the fact that the last refill of soap and chlorine provided at discharge was for a period of 1 month only and that the weekly promotion sessions stopped after discharge, affecting the level of WASH-related good practices. Provision of soap and chlorine tablets as well as hygiene promotion sessions could be more beneficial if sustained for at least 2 months after discharge, and included the promotion of alternative methods for water treatment such as solar disinfection or filtration. Relapse rate was higher at 2 than at 6 months after discharge in both groups, as seen in previous studies.^14,17^ Furthermore, we did not achieve the required sample size at 2-month follow-up as only 1,107 children were visited instead of the 1,400 calculated. Reasons for not reaching the total sample size included shortage in RUTF, lower recruitment rate than expected, and shortage of funds. Overall, however, the absolute difference was smaller than expected. The study sample was not powered to find such a small difference. The results should therefore be confirmed by future bigger studies.

Our results reported an increased recovery rate in the intervention group. This result was based on clinical judgment but was confirmed in the sensitive analysis based on anthropometric data, excluding any potential misclassification bias. Difference in the total recovery rates between the two analyses highlighted some difficulties for field staff to adhere strictly to the SAM protocol in routine practice. The causal mechanism leading to increased recovery rate in the intervention group is not straightforward to identify. One possibility is through a reduced duration of infectious disease episodes;^3,4,24^ yet in our study, we observed a shorter duration of diarrhea and vomiting, although the reduction in diarrhea was not statistically different between groups. A Cochrane review in 2006 did, however, conclude that water treatment at point-of-use may reduce diarrhea by one-quarter (chlorine tablets) to one-third (flocculation and disinfection sachets).^25^ Still, other pathways in the fecal-oral transmission routes remain. Latrine use is limited in the study area and a large part of the population still practices open defecation. This can provide another source of contamination that offsets the gains of safe water provision and soap. The process indicators also showed that practices improved but without reaching a high level of compliance. The adoption of improved practices might just not have been enough to show a difference in longitudinal diarrhea prevalence. The relatively small number of reported diarrhea cases in the whole group was unexpected and may have diluted the intervention effect. Underreporting is classically seen for self-reported morbidity and might have affected all participants in our study.^26–29^ Indeed, both groups received basic hygiene education and care practice sessions. Furthermore, study staff who provided these sessions was different from the HC staff who collected information about child morbidity. This low longitudinal prevalence could also explain the nonsignificant increase in weight gain velocity, which has been reported to be strongly associated with diarrhea longitudinal prevalence in children.^22^

The observed reduction in the time-to-recovery is in line with a study conducted in the Democratic Republic of Congo, where the use of flocculent-disinfectant for water was linked to a 4-day reduction in the time-to-recovery.^30^ This reduction would reduce the costs, both for the program and the beneficiaries. However, this result should be taken with caution, as it is not possible to exclude a misclassification bias due to the staff judgment. The sensitive analysis reported a nonsignificant difference, which could also be due to a lack of power.

Finally, our results showed that although the household WASH package was well accepted and used, there is still potential for improvement and therefore potential in its effect. Indeed, self-reported practices often overestimate true practices because of social desirability bias. Although it was reported that more than 80% used the correct chlorination procedure at the second visit, the correct level of residual chlorine in the water was only reported in 50% of the households. We cannot exclude that other non-WASH–related behaviors (e.g., child feeding practices) may have accounted for the improved recovery. This particularly indicates the need to improve the promotion component when delivering the kit. The storage container was correctly used in only 50% of the households. Containers with a tap would be a solution to avoid contamination of water when chlorine residual is < 0.5 mg/L. Alternatively, an increased chlorine dosage (without compromising acceptance) could maintain an adequate concentration for a longer duration.^31^

The shortage in RUTF during the study period was a potential bias. However, our results showed that both groups were equally affected. Furthermore, all models were adjusted for the number of visits with shortage. The way diarrhea was defined excludes more chronic forms of enteropathies with less frequent passages of stools, such as *Giardia*^32,33^ or environmental enteric dysfunctions (EED).^34^ We cannot exclude that these may have affected recovery rates. Easy-to-use diagnostic tools for EED are necessary if we want to better interpret WASH intervention pathways. Unfortunately, logistical and organizational constraints in our setting did not allow us to conduct more advanced etiological diagnostics on diarrhea. Further studies should include this component. It is also to be mentioned that the observed positive results were obtained after two home visits in the intervention group, which is not done in standard “WASH in Nut” interventions.

In the Sahel context, the provision of a household WASH package increased the recovery rate among children treated for noncomplicated SAM and decreased their time-to-recovery. This study shows that the integrated “WASH in Nut” approach has the potential to increase program performance. We recommend further robust trials in other settings to confirm our results. In the meantime, our study can inform health authorities in settings where the WASH component is not yet integrated into the nutritional programs.
